# Triage body temperature and its influence on patients with acute myocardial infarction

**DOI:** 10.1186/s12872-023-03372-y

**Published:** 2023-08-04

**Authors:** Shih-Hao Chen, Hung-Chieh Chang, Po-Wei Chiu, Ming-Yuan Hong, I-Chen Lin, Chih-Chun Yang, Chien-Te Hsu, Chia-Wei Ling, Ying-Hsin Chang, Ya-Yun Cheng, Chih-Hao Lin

**Affiliations:** 1 Department of Emergency Medicine, College of Medicine, National Cheng Kung University Hospital, National Cheng Kung University, Tainan, 70403 Taiwan; 2 School of Medicine, College of Medicine, National Sun Yat-sen University, 804, No.70, Lien-hai Rd, Kaohsiung, 804 Taiwan

**Keywords:** Myocardial infarction, Emergency department, Triage, Body temperature, In-hospital cardiac arrest

## Abstract

**Background:**

Fever can occur after acute myocardial infarction (MI). The influence of body temperature (BT) after hospital arrival on patients with acute MI has rarely been investigated.

**Methods:**

Patients who were diagnosed with acute MI in the emergency department (ED) of a tertiary teaching hospital between 1 January 2020 and 31 December 2020 were enrolled. Based on the tympanic temperature obtained at the ED triage, patients were categorized into normothermic (35.5°*C*-37.5°*C*), hypothermic (< 35.5°*C*), or hyperthermic (> 37.5°*C*) groups. The primary outcome was in-hospital cardiac arrest (IHCA), while the secondary outcomes were adverse events. Statistical significance was set at p < 0.05.

**Results:**

There were 440 enrollees; significant differences were found among the normothermic (n = 369, 83.9%), hypothermic (n = 27, 6.1%), and hyperthermic (n = 44, 10.0%) groups in the triage respiratory rate (median [IQR]) (20.0 [4.0] cycles/min versus 20.0 [4.0] versus 20.0 [7.5], p = 0.009), triage heart rate (88.0 [29.0] beats/min versus 82.0 [28.0] versus 102.5 [30.5], p < 0.001), presence of ST-elevation MI (42.0% versus 66.7% versus 31.8%, p = 0.014), need for cardiac catheterization (87.3% versus 85.2% versus 72.7%, p = 0.034), initial troponin T level (165.9 [565.2] ng/L versus 49.1 [202.0] versus 318.8 [2002.0], p = 0.002), peak troponin T level (343.8 [1405.9] ng/L versus 218.7 [2318.2] versus 832.0 [2640.8], p = 0.003), length of ICU stay (2.0 [3.0] days versus 3.0 [8.0] versus 3.0 [9.5], p = 0.006), length of hospital stay (4.0 [4.5] days versus 6.0 [15.0] versus 10.5 [10.8], p < 0.001), and infection during hospitalization (19.8% versus 29.6% versus 63.6%, p < 0.001) but not in IHCA (7.6% versus 14.8% versus 11.4%, p = 0.323) or any adverse events (50.9% versus 48.1% versus 63.6%, p = 0.258). Multivariable analysis showed no significant association of triage BT with IHCA or any major complication.

**Conclusion:**

Triage BT did not show a significant association with IHCA or adverse events in patients with acute MI. However, triage BT could be associated with different clinical presentations and should warrant further investigation.

**Supplementary Information:**

The online version contains supplementary material available at 10.1186/s12872-023-03372-y.

## Background

Fever can occur after acute myocardial infarction (MI) [1, 2]. Body temperature could increase by more than 1 °C as soon as the first 4 to 8 h after onset of symptoms, peak in the first to second day with an average of 37.5 °C, and decrease in the fourth to fifth day after admission [2]. The development of fever involves an intricate response of the human pathophysiological pathway. After MI, monocytes and lymphocytes infiltrate the myocardium [3, 4]. Cytokines, including interleukin (IL)-1, IL-6, IL-8, tumor necrosis factor-α, and interferon-γ, are released and pass through the blood‒brain barrier to influence the temperature regulatory center in the hypothalamus [5]. Prostaglandin E2, which is released afterward, plays an essential role in the development of fever.

Elevation of body temperature influences the biochemistry and clinical outcomes of myocardial infarction patients [6–11]. Fever not only triggers an inflammatory response but also aggravates atherosclerosis and causes a “no-flow phenomenon” in the coronary artery, leading to an increase in infarction size [6]. Body temperature elevation is associated with increases in white blood cell count (WBC), C-reactive protein (CRP) and peak creatine kinase (CK) in ST-elevation MI (STEMI) patients [7–9]. Cardiac magnetic resonance imaging showed that myocardial infarction size is larger in the febrile group than in the nonfebrile group [10]. Moreover, higher rates of heart failure, cardiogenic shock, one-year readmission, and one-year in-stent thrombosis were reported in patients with MI who had elevated body temperature [11, 12]. On the other hand, lower body temperature is also associated with inferior outcomes in patients in the emergency department (ED) [13]. Hypothermia can cause hypokalemia at first and then hyperkalemia due to cell lysis and depolarization [14]. However, therapeutic hypothermia has been proposed for the treatment of acute MI to reduce the damage caused by post-ischemia–reperfusion injury syndrome [15].

Most of the studies that explored the association of body temperature and MI focused on the body temperature recorded after admission to the intensive care unit [7, 9–11]. To our knowledge, the effect of triage body temperature on patients with acute MI has rarely been investigated before. The ED triage system uses basic information, including blood pressure, respiratory rates, oxygen saturation, and body temperature, to classify and prioritize patients. The initial body temperature could be associated with mortality in certain patient groups [16]. This study aimed to investigate the influence of triage body temperature in patients with acute MI.

## Methods

### Data source and study population

This study was a retrospective study of consecutive patients with acute MI from January 1, 2020, to December 31, 2020, in the ED of a tertiary medical center in Tainan, Taiwan. The average number of ED visits is 100,668 annually. Data were retrospectively retrieved from electronic medical records and were deidentified for analysis. There was no community outbreak of COVID-19 in Tainan city during the study period [17, 18].

Body temperature was measured as tympanic temperature by an infrared ear thermometer (Chang Gung infrared-thermometer^@^, Chang Gung Medical Technology Co., Taoyuan, Taiwan), which was calibrated regularly. The tympanic temperature, which is a good index of core temperature, is usually 0.3–0.6 °C higher than an oral temperature [19]. We used the tympanic temperature, instead of the oral or rectal temperatures, because the tympanic temperature is widely utilized in the adult emergency department, especially the triage system [20]. Patients who had a triage body temperature greater than 37.5 °C were assigned to the hyperthermic group [8, 12, 21]. Those who had a triage body temperature less than 35.5 °C were assigned to the hypothermic group [22]. Those who had a triage BT between 35.5 and 37.5 °C were assigned to the normothermic group.

The diagnosis of acute MI was based on the Fourth Universal Definition of Myocardial Infarction Consensus Document [23]. Patients were diagnosed with STEMI or non-ST-elevation MI (NSTEMI) if they had a troponin level above the 99th percentile of the upper reference limit and at least one of the following: symptoms of myocardial ischemia, new ischemic changes on electrocardiogram, development of pathologic Q waves, imaging evidence of new loss of myocardium or new regional wall motion abnormality in a pattern consistent with ischemia, or identification of thrombus by angiography or autopsy. Patients were excluded if they were younger than 20 years or pregnant; had out-of-hospital cardiac arrest, trauma, or autoimmune disease; or were missing triage body temperature values.

### The normothermic, hypothermic, and hyperthermic groups

#### Basic characteristics

Basic characteristics consisted of age, sex, triage measurements of vital signs, peak body temperature within 24 h of arrival, comorbidities, intervention records, and a primary diagnosis of STEMI or NSTEMI. Triage measurements of vital signs included body temperature, respiratory rate, systolic blood pressure, diastolic blood pressure, mean blood pressure and saturation.

Comorbidities included coronary artery disease (CAD), diabetes mellitus (DM), hypertension (HTN), heart failure (HF), chronic kidney disease (CKD), cerebral vascular accident (CVA), malignancy, and dyslipidemia. The biochemical data included WBC, serum creatinine, CRP, and the initial and peak troponin-T levels. The clinical stratification of the Killip class and syntax score [24], time to cardiac catheterization or intervention, development of infection that required antibiotic treatment, initial blood culture results from emergency department or admission, length of stay in hospital, and length of stay in the intensive care unit (ICU) were also retrieved for analysis.

#### Outcome definition

The primary outcome was in-hospital cardiac arrest. The secondary outcomes were major adverse events, which included shock; pulmonary edema; new heart failure; arrhythmias such as ventricular tachycardia, ventricular fibrillation, or new atrial fibrillation; major bleeding that required transfusion; or acute renal failure, which was defined as a deteriorating glomerular filtration rate less than 15 ml/min/1.73 m^2^ or a need for hemodialysis. Patients who required antibiotic treatment per clinical judgment during hospitalization were defined as having infections.

#### Statistical analyses

Continuous variables are presented as the median and interquartile range (IQR). Categorical variables are presented as percentages. Student’s t test or one-way ANOVA was conducted to determine differences in continuous variables between groups. The Mann‒Whitney U test or Kruskal‒Wallis H test, which is a rank-based nonparametric test, was used to identify statistically significant differences between two or more groups for an independent variable and a nonnormally distributed continuous dependent variable. Bonferroni’s post-hoc 2 by 2 comparison tests were used when one-way ANOVA or the Kruskal‒Wallis H test indicated statistical significance. Chi-square analysis or Fisher’s exact test was performed to determine differences in categorical variables between groups, as applicable. We did not impute missing values, and all missing values were regarded as blank and excluded from the final analysis.

To identify the independent risk factors for outcome, we used univariable logistic regression followed by multivariable regression to obtain the associated odds ratios (ORs) and their 95% confidence intervals (CIs). In the multivariable analyses, we constructed full models that included triage BT and all the potential risk factors identified in the univariable analyses and then constructed the final models by applying the backward stepwise approach (exclusion set as p > 0.15) and medical knowledge in accordance with the hypothesis. We also tried to apply propensity matching with baseline characteristics and comorbidities for patients with IHCA.

Statistical analyses were performed with MS. EXCEL and SPSS 17.0 statistical software (SPSS Inc., Chicago, IL, USA). All statistical tests were performed at a two-sided significance level of 0.05.

## Results

**Fig. 1:**
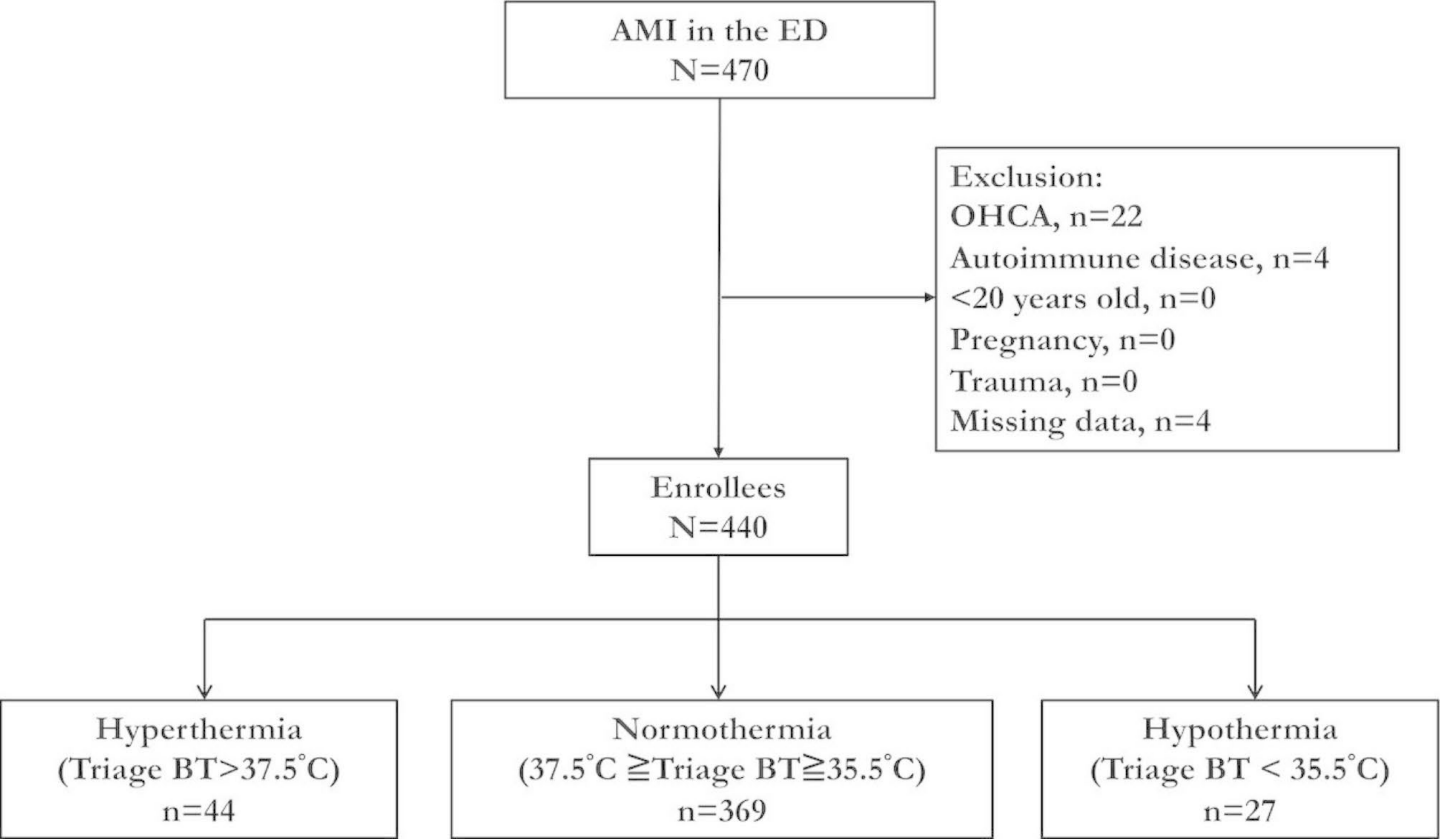
Patient inclusion diagram. Abbreviations: AMI, acute myocardial infarction; BT, body temperature; ED, emergency department; OHCA, out-of-hospital cardiac arrest

The inclusion and exclusion diagram is shown in Fig. 1. There were 470 patients with acute MI in the ED during the study period, and 30 patients were excluded due to out-of-hospital cardiac arrest (n = 22), autoimmune diseases (n = 4), and missing records of triage body temperature (n = 4). Among the 440 patients who were enrolled in the final analysis, 369 (83.9%) were classified into the normothermic group (37.5 °C ≥ triage body temperature ≥ 35.5 °C), 27 (6.1%) were classified into the hypothermic group (triage body temperature < 35.5 °C), and 44 (10.0%) were classified into the hyperthermic group (triage body temperature > 37.5 °C). The distribution of triage body temperature is depicted in Supplementary Figure S1.

The basic characteristics of the enrollees are shown in Table 1. The normothermic group, hypothermic group, and hyperthermic group demonstrated statistically significant differences regarding triage body temperature (°C) (median [IQR]) (36.5 [0.8], 35.3 [0.4], and 37.9 [0.7], p < 0.001), peak body temperature within 24 h of ED arrival (°C) (37.1 [0.6], 36.9 [0.7], and 38.1 [1.0], p < 0.001), triage respiratory rate (cycles/min) (20.0 [4.0], 20.0 [4.0], and 20.0 [7.5], p = 0.009), triage heart rate (beats/min) (88.0 [29.0], 82.0 [28.0], and 102.5 [30.5], p < 0.001), the presence of ST-elevation MI (42.0%, 66.7%, and 31.8%, p = 0.014), and the need for cardiac catheterization (87.3%, 85.2%, and 72.7%, p = 0.034), positive blood cultures in STEMI (1.3%, 5.6% and 7.1%, p = 0.201), positive blood cultures in NSTEMI (2.3%, 11.1% and 10%, p = 0.048). The hyperthermic group showed a significantly higher respiratory rate and heart rate than the other groups in multiple comparison analysis.

**Table 1:** Basic characteristics among different triage body temperature groups

	Normothermic (N = 369) n (%) or median (IQR)	Hypothermic (N = 27) n (%) or median (IQR)	Hyperthermic (N = 44) n (%) or median (IQR)	p value Kruskal‒Wallis	Post Hoc Bonferroni
Age (y/o)	67.8 (21.7)	69.9 (14.2)	72.3 (24.8)	0.260	
Sex: Male	269 (72.9%)	21 (77.8%)	28 (63.6%)	0.347	
Triage BT (°C)	36.5 (0.8)	35.3 (0.4)	37.9 (0.7)	< 0.001	^#, $, &^
Peak BT in 24 h (°C)	37.1 (0.6)	36.9 (0.7)	38.1 (1.0)	< 0.001	^$, &^
Triage					
RR (rate/min)	20.0 (4.0)	20.0 (4.0)	20.0 (7.5)	0.009	^$, &^
SBP (mmHg)	140.0 (46.0)	125.0 (56.0)	137.5 (43.0)	0.293	
DBP (mmHg)	84.0 (28.5)	74.0 (42.0)	79.5 (23.8)	0.058	
MBP (mmHg)	102.3 (31.7)	89.0 (45.3)	102.3 (28.4)	0.089	
HR (beat/min)	88.0 (29.0)	82.0 (28.0)	102.5 (30.5)	< 0.001	^$, &^
SpO_2_ (%)	98.0 (5.0)	99.0 (2.8)	98.0 (6.0)	0.598	
Comorbidity					
CAD	94 (25.5%)	7 (25.9%)	10 (22.7%)	0.921	
DM	168 (45.5%)	10 (37.0%)	19 (43.2%)	0.676	
HTN	240 (65.0%)	12 (44.4%)	28 (63.6%)	0.100	
HF	45 (12.2%)	2 (7.4%)	3 (6.8%)	0.455	
CKD	102 (27.6%)	8 (29.6%)	8 (18.2%)	0.385	
Old CVA	46 (12.5%)	3 (11.1%)	11 (25.0%)	0.067	
Malignancy	33 (8.9%)	3 (11.1%)	6 (13.6%)	0.581	
Dyslipidemia	110 (29.8%)	8 (29.6%)	12 (27.3%)	0.941	
PCI	322 (87.3%)	23 (85.2%)	32 (72.7%)	0.034	
Door to catheterization (min) (N = 341)	110.0 (841.0)	78.0 (394.0)	701.0 (1358.0)	0.101	
Positive blood culture	7 (1.9%)	2 (7.4%)	4 (9.1%)	0.011	
STEMI	155 (42.0%)	18 (66.7%)	14 (31.8%)	0.014	
NSTEMI	214 (58.0%)	9 (33.3%)	30 (68.2%)	0.014	
STEMI, positive blood culture	2 (1.3%)	1 (5.6%)	1 (7.1%)	0.201	
NSTEMI, positive blood culture	5 (2.3%)	1 (11.1%)	3 (10%)	0.048	

^#^ Normothermic vs. Hypothermic, ^$^ Normothermic vs. Hyperthermic, ^&^ Hypothermic vs. Hyperthermic

Abbreviations: BT, body temperature; CAD, coronary artery disease; CKD, chronic kidney disease; CVA, cerebral vascular accident; DBP, diastolic blood pressure; DM, diabetes mellitus; HF, heart failure; HR, heart rate; HTN, hypertension; IQR, interquartile range; MBP, mean blood pressure; NSTEMI, non-ST-elevation myocardial infarction; PCI, percutaneous coronary intervention; RR, respiratory rate; SBP, systolic blood pressure; STEMI, ST-elevation myocardial infarction

The correlations of triage body temperature with biochemical values and clinical outcomes are displayed in Table 2. The normothermic group, hypothermic group, and hyperthermic group showed statistically significant differences regarding initial troponin T level (ng/L) (165.9 [565.2], 49.1 [202.0], and 318.8 [2002.0], p = 0.002), peak troponin T level (ng/L) (343.8 [1405.9], 218.7 [2318.2], and 832.0 [2640.8], p = 0.003), infection (19.8%, 29.6%, and 63.6%, p < 0.001), length of ICU stay (days) (2.0 [3.0], 3.0 [8.0], and 3.0 [9.5], p = 0.006), and length of hospital stay (days) (4.0 [4.5], 6.0 [15.0], and 10.5 [10.8], p < 0.001).

**Table 2:** Biochemical values and clinical outcome

	Normothermic (N = 369) n (%) or median (IQR)	Hypothermic (N = 27) n (%) or median (IQR)	Hyperthermic (N = 44) n (%) or median (IQR)	p value ANOVA K-W test	Post Hoc Bonferroni
WBC (10^^3^/*µL*)	10.0 (4.8)	9.7 (5.1)	12.1 (6.1)	0.144	
Creatinine	1.0 (0.8)	1.1 (1.7)	1.0 (0.6)	0.637	
Initial TnT (ng/L)	165.9 (565.2)	49.1 (202.0)	318.8 (2002.0)	0.002	^#, &^
Peak TnT (ng/L)	343.8 (1405.9)	218.7 (2318.2)	832.0 (2640.8)	0.003	^$, &^
CRP (mg/L) (N = 15)	4.1 (111.9)	5.4 (7.0)	96.8 (87.5)	0.259	
Killip class (N = 179)	1.0 (2.0)	2.0 (3.0)	3.0 (2.0)	0.069	
Syntax score (N = 168)	20.0 (21.0)	23.0 (31.0)	23.5 (30.3)	0.732	
Infection	73 (19.8%)	8 (29.6%)	28 (63.6%)	< 0.001	^$, &^
ICU stay (days)	2.0 (3.0)	3.0 (8.0)	3.0 (9.5)	0.006	^#^
Hospital stay (days)	4.0 (4.5)	6.0 (15.0)	10.5 (10.8)	< 0.001	^$^

^#^ Normothermic vs. Hypothermic, ^$^ Normothermic vs. Hyperthermic, ^&^ Hypothermic vs. Hyperthermic

Abbreviations: CRP, C-reactive protein; ICU, intensive care unit; IQR, interquartile range; TnT, troponin-T; WBC, white blood cell

The adverse events and complications are shown in Table 3. The normothermic group, hypothermic group, and hyperthermic group showed statistically significant differences in infection during hospitalization (19.8%, 29.6%, and 63.6%, p < 0.001) and acute renal failure (7.9%, 22.2%, and 15.9%, p = 0.016) but not in in-hospital cardiac arrest (7.6%, 14.8%, and 11.4%, p = 0.323) or overall complications (50.9%, 48.1%, and 63.6%, p = 0.258).

**Table 3:** Incidence of adverse outcomes and infection based on triage body temperature

	Normothermic (N = 369) n (%)	Hypothermic (N = 27) n (%)	Hyperthermic (N = 44) n (%)	p value
Overall complication*	188 (50.9%)	13 (48.1%)	28 (63.6%)	0.258
In-hospital cardiac arrest	28 (7.6%)	4 (14.8%)	5 (11.4%)	0.323
Shock	46 (12.5%)	5 (18.5%)	9 (20.5%)	0.258
Pulmonary edema	94 (25.5%)	5 (18.5%)	15 (34.1%)	0.311
New HF	64 (17.3%)	6 (22.2%)	14 (31.8%)	0.063
VT, Vf or new Af	68 (18.4%)	8 (29.6%)	9 (20.5%)	0.356
Major bleeding	20 (5.4%)	2 (7.4%)	1 (2.3%)	0.588
Acute renal failure	29 (7.9%)	6 (22.2%)	7 (15.9%)	0.016

Abbreviations: Af, atrial fibrillation; HF, heart failure; Vf, ventricular fibrillation; VT, ventricular tachycardia

* Overall complication included all listed adverse events

The main causes of IHCA were showed in Supplementary Table 1. No significant differences were observed regarding the causes of IHCA among the groups.

The risk factors for in-hospital cardiac arrest are shown in Table 4. At the end of follow-up, we identified 37 (8.4%) patients with in-hospital cardiac arrest. The total sample size included into the full and final model analysis was 424 from the original 440 patients. The missing rate was 3.6% and therefore we did not interpolate data into the missing one. The significant risk factors for cardiac arrest from univariable logistic regression included age, MDP, oxygen saturation, DM, and coronary intervention. In the full or final models, which included all the potential risk factors and add-on STEMI/NSTEMI for cardiac arrest, the adjusted OR (AOR) associated with triage body temperature was not significant but was still included in the final model. In the final model, we demonstrated the BT factors with 1. 74 (95% CI: 0.50–6.03, p = 0.382) in the hypothermic group, and 1.26 (95% CI: 0.42–3.78, p = 0.687) in the hyperthermic group. Other independent risk factors for cardiac arrest included age (AOR = 1.04, 95% CI: 1.00-1.07, p = 0.027), MBP (AOR = 0.97, 95% CI: 0.96–0.99, p < 0.001), DM (AOR = 2.49, 95% CI: 1.14–5.45, p = 0.022), receiving cardiac catheterization (AOR = 0.24, 95% CI: 0.10–0.58, p = 0.002), and STEMI (AOR = 2.49, 95% CI: 1.09–5.69, p = 0.031)/NSTEMI (AOR = 0.40, 95% CI: 0.18–0.92, p = 0.031).

**Table 4:** The incidence and risk factors for in-hospital cardiac arrest

	Not IHCA (N = 403) n (%) or median (IQR)	IHCA (N = 37) n (%) or median (IQR)	p value Mann-Whitney	OR (95% CI)^a^	AOR (95% CI)^b^	AOR (95% CI)^c^
Age (y/o)	67.5 (20.4)	77.6 (16.2)	< 0.001	1.05 (1.02–1.08)***	1.03 (1.00-1.07)*	1.04 (1.00-1.07)*
Sex: Male	292 (72.5%)	26 (70.3%)	0.776	0.90 (0.43–1.88)		
Triage BT (*°C*)	36.6 (0.9)	36.7 (1.5)	0.965	1.03 (0.69–1.54)		
Normothermic	341 (84.6%)	28 (75.7%)	0.323	1.00	1.00	1.00
Hypothermic	23 (5.7%)	4 (10.8%)		2.12 (0.69–6.55)	2.17 (0.59–7.89)	1.74 (0.50–6.03)
Hyperthermic	39 (9.7%)	5 (13.5%)		1.56 (0.57–4.28)	0.98 (0.30–3.26)	1.26 (0.42–3.78)
Peak BT in 24 h (*°C*)	37.2 (0.7)	37.1 (1.1)	0.432	1.04 (0.64–1.70)		
RR (rate/min)	20.0 (4.0)	22.0 (7.0)	0.017	1.04 (0.99–1.09)		
SBP (mmHg)	141.0 (44.3)	125.0 (47.0)	< 0.001	0.98 (0.97–0.99)***		
DBP (mmHg)	84.5 (27.3)	68.0 (32.0)	< 0.001	0.96 (0.95–0.98)***		
MBP (mmHg)	103.0 (31.7)	88.7 (36.0)	< 0.001	0.97 (0.96–0.99)***	0.97 (0.95–0.98)***	0.97 (0.96–0.99)***
HR (beat/min)	88.0 (29.3)	95.0 (42.0)	0.424	1.00 (0.98–1.02)		
SpO_2_ (%)	99.0 (5.0)	97.0 (8.3)	0.011	0.95 (0.91–0.99)*	0.99 (0.93–1.04)	
CAD	100 (24.8%)	11 (29.7%)	0.510	1.28 (0.61–2.69)		
DM	172 (42.7%)	25 (67.6%)	0.004	2.80 (1.37–5.73)**	2.11 (0.95–4.73)	2.49 (1.14–5.45)
HTN	257 (63.8%)	23 (62.2%)	0.846	0.93 (0.47–1.87)		
HF	44 (10.9%)	6 (16.2%)	0.331	1.58 (0.62–3.99)		
CKD	105 (26.1%)	13 (35.1%)	0.233	1.54 (0.76–3.13)		
Old CVA	52 (12.9%)	8 (21.6%)	0.139	1.86 (0.81–4.29)		
Malignancy	38 (9.4%)	4 (10.8%)	0.784	1.16 (0.39–3.46)		
Dyslipidemia	118 (29.3%)	12 (32.4%)	0.688	1.16 (0.56–2.38)		
PCI	353 (87.6%)	24 (64.9%)	< 0.001	0.26 (0.13–0.55)***	0.24 (0.09–0.61)**	0.24 (0.10–0.58)**
Door to catheterization (min) (N = 341)	120.0 (846.0)	83.0 (787.8)	0.283	1.00 (1.00–1.00)		
STEMI	168 (41.7%)	19 (51.4%)	0.255	1.48 (0.75–2.89)	1.96 (0.83–4.64)	2.49 (1.09–5.69)*
NSTEMI	235 (58.3%)	18 (48.6%)	0.255	0.68 (0.35–1.33)	0.51 (0.22–1.20)	0.40 (0.18–0.92)

*p < 0.05, **p < 0.01, ***p < 0.001 / ^a^ single variable, ^b^ full model, ^c^ final model

Abbreviations: BT, body temperature; CAD, coronary artery disease; CI, confidence interval; CKD, chronic kidney disease; CVA, cerebral vascular accident; DBP, diastolic blood pressure; DM, diabetes mellitus; HF, heart failure; HR, heart rate; HTN, hypertension; IHCA, in-hospital cardiac arrest; IQR, interquartile range; MBP, mean blood pressure; NSTEMI, non-ST-elevation myocardial infarction; OR, odds ratio; PCI, percutaneous coronary intervention; RR, respiratory rate; SBP, systolic blood pressure; STEMI, ST-elevation myocardial infarction

We tried to apply propensity matching with baseline characteristics and comorbidities for patients with IHCA. There was with a tolerance score of 0.02 and without replacement; and the selected matching were 37 pairs of fuzzy matches (4.501% of 822 tries). Nevertheless, the AOR in the model was still difficult to be perfect adjusted and modified. The results of propensity matching analysis were provided in Supplementary Table 2.

The risk factors for complications are shown in Table 5. We identified 229 (52.0%) patients with complications. The significant risk factors for any complication from the univariable logistic regression included age, sex, triage body temperature, respiratory rate, heart rate, oxygen saturation, CAD, DM, HTN, HF, CKD, CVA, and cardiac catheterization. In the final model, which included all the potential risk factors and STEMI/NSTEMI for any complication, the AOR associated with triage body temperature were 0.84 (95% CI: 0.32–2.23, p = 0.728) in hypothermic group, and 1.03 (95% CI: 0.45–2.33, p = 0.948) in hyperthermic group. In the final model, the AOR associated with triage body temperature was not significant but was still be included in the model. Other independent risk factors for complications included age (AOR = 1.06, 95% CI: 1.04–1.08, p < 0.001), respiratory rate (AOR = 1.07, 95% CI: 1.00-1.14, p = 0.047), heart rate (AOR = 1.02, 95% CI: 1.01–1.03, p = 0.005), oxygen saturation (AOR = 0.87, 95% CI: 0.82–0.94, p < 0.001), CKD (AOR = 1.99, 95% CI: 1.13–3.49, p = 0.016), and STEMI (AOR = 1.65, 95% CI: 1.00-2.70, p = 0.048)/NSTEMI (AOR = 0.61, 95% CI: 0.37–0.99, p = 0.048).

**Table 5:** The incidence and risk of complications

	Without complications (N = 211) n (%) or median (IQR)	With complications (N = 229) n (%) or median (IQR)	p value Mann-Whitney	OR (95% CI)^a^	AOR (95% CI)^b^	AOR (95% CI)^c^
Age (y/o)	62.4 (17.6)	75.9 (18.9)	< 0.001	1.07 (1.05–1.09)***	1.06 (1.04–1.08)***	1.06 (1.04–1.08)***
Sex: Male	165 (78.2%)	153 (66.8%)	0.016	0.56 (0.37–0.86)**	1.15 (0.66-2.00)	
Triage BT (*°C*)	36.5 (0.9)	36.7 (1.1)	0.016	1.28 (1.01–1.61)*		
Normothermic	181 (85.8%)	188 (82.1%)	0.258	1.00	1.00	1.00
Hypothermic	14 (6.6%)	13 (5.7%)		0.89 (0.41–1.95)	0.83 (0.31–2.22)	0.84 (0.32–2.23)
Hyperthermic	16 (7.6%)	28 (12.2%)		1.69 (0.88–3.22)	1.01 (0.44–2.29)	1.03 (0.45–2.33)
Peak BT in 24 h (*°C*)	37.1 (0.7)	37.2 (0.9)	0.048	1.35 (1.01–1.80)*		
RR (rate/min)	20.0 (2.0)	20.0 (6.0)	< 0.001	1.22 (1.14–1.29)***	1.07 (1.00-1.15)*	1.07 (1.00-1.14)*
SBP (mmHg)	144.0 (36.0)	136.5 (56.8)	0.046	0.99 (0.99-1.00)		
DBP (mmHg)	86.0 (28.0)	80.0 (29.0)	0.036	0.99 (0.98-1.00)		
MBP (mmHg)	103.3 (28.3)	99.0 (34.3)	0.021	0.99 (0.98-1.00)		
HR (beat/min)	82.0 (22.0)	95.0 (32.8)	< 0.001	1.02 (1.01–1.03)***	1.02 (1.00-1.03)**	1.02 (1.01–1.03)**
SpO_2_ (%)	99.0 (2.0)	97.0 (8.0)	< 0.001	0.82 (0.77–0.88)***	0.88 (0.82–0.94)***	0.87 (0.82–0.94)***
CAD	42 (19.9%)	69 (30.1%)	0.014	1.74 (1.12–2.70)*	1.34 (0.74–2.42)	
DM	74 (35.1%)	123 (53.7%)	< 0.001	2.15 (1.46–3.15)***	1.03 (0.62–1.72)	
HTN	120 (56.9%)	160 (69.9%)	0.005	1.76 (1.19–2.60)**	1.03 (0.62–1.72)	
HF	17 (8.1%)	33 (14.4%)	0.036	1.92 (1.04–3.56)*	0.71 (0.31–1.62)	
CKD	33 (15.6%)	85 (37.1%)	< 0.001	3.18 (2.01–5.03)***	1.95 (1.05–3.64)*	1.99 (1.13–3.49)*
Old CVA	19 (9.0%)	41 (17.9%)	0.007	2.20 (1.23–3.94)**	1.00 (0.49–2.06)	
Malignancy	17 (8.1%)	25 (10.9%)	0.308	1.40 (0.73–2.67)		
Dyslipidemia	65 (30.8%)	65 (28.4%)	0.578	0.89 (0.59–1.34)		
PCI	194 (91.9%)	183 (79.9%)	< 0.001	0.35 (1.93 − 0.63)***	0.83 (0.38–1.79)	
Door to catheterization (min) (N = 341)	130.5 (845.5)	101.0 (844.5)	0.475	1.00 (1.00–1.00)		
STEMI	97 (46.0%)	90 (39.3%)	0.157	0.76 (0.52–1.11)	1.70 (1.02–2.83)*	1.65 (1.00-2.70)*
NSTEMI	114 (54.0%)	139 (60.7%)	0.157	1.31 (0.90–1.92)	0.59 (0.35–0.98)*	0.61 (0.37–0.99)*

*p < 0.05, **p < 0.01, ***p < 0.001; ^a^ single variable, ^b^ full model, ^c^ final model

Abbreviations: BT, body temperature; CAD, coronary artery disease; CI, confidence interval; CKD, chronic kidney disease; CVA, cerebral vascular accident; DBP, diastolic blood pressure; DM, diabetes mellitus; HF, heart failure; HR, heart rate; HTN, hypertension; IHCA, in-hospital cardiac arrest; IQR, interquartile range; MBP, mean blood pressure; NSTEMI, non-ST-elevation myocardial infarction; OR, odds ratio; PCI, percutaneous coronary intervention; RR, respiratory rate; SBP, systolic blood pressure; STEMI, ST-elevation myocardial infarction

## Discussion

The results of our study showed that the triage body temperature of patients with acute MI was associated with the levels of certain biochemical parameters, length of hospital stay, and development of infection or acute renal failure in the hospital course, but not in-hospital cardiac arrest or overall complications. Our study also identified several risk factors that may serve as warning signs for cardiac arrest and complications.

In our study, the different triage body temperature groups shared similar basic characteristics despite minor abnormalities. The respiratory rate and heart rate were higher in the hyperthermic group, which was consistent with previous studies [25]. The hyperthermic group had fewer coronary interventions. The reasons for not undergoing coronary intervention included patient refusal, alternative intervention such as coronary artery bypass surgery, or unsuitability for intervention. The higher rates of NSTEMI or infection in the hyperthermic group might have led to fewer patients in this group undergoing coronary intervention.

Accidental hypothermia was reported in patients with MI. Hypothermia can cause profound cellular damage, especially in skeletal and cardiac muscle [26]. The rise and fall of cardiac enzymes, especially the CK-myocardial band, can occur in hypothermic patients without MI [27, 28]. Most patients with confirmed myocardial infarction in our study underwent coronary intervention. The hypothermic group showed lower respiratory rates and heart rates than the hyperthermic group and lower diastolic blood pressure than the normothermic group. A systolic blood pressure less than 90 mmHg is a significant risk factor for mortality in patients with hypothermia [29]. However, the hypothermic group in this study consisted of patients with mild-to-moderate hypothermia with triage body temperatures ranging from 32.8 to 35.4 °C and did not demonstrate significantly lower systolic blood pressure.

The level of cardiac enzymes is correlated with the severity of acute MI [30]. Previous studies found that patients with STEMI who had a higher peak body temperature could have a higher level of CK [8, 11]. In this study, we found that the initial and peak troponin T levels were higher even in the hypothermic and hyperthermic groups.

The hyperthermic group had a longer door-to-catheterization time. There was no community outbreak of COVID-19 during the study period [17, 18] and none of the patients that were included in this study had COVID infection. We suspected that the presence of hyperthermia may have the clinicians to take precautions and perform more laboratory examination or imaging studies. The ambiguity of presenting symptoms also renders more time before making a diagnosis of myocardial infarction.

Among the 109 patients who had infections that required antibiotic treatment during hospitalization, pneumonia (59.6%) was the leading cause of infection, followed by urinary tract infection (34.9%) and soft tissue infection (1.8%). Furthermore, among the 65 patients who were diagnosed with pneumonia, the positive rates of sputum and blood culture were 24.6% and 6.2%, respectively, which were lower than those in previous reports [31, 32]. We suspected that pneumonia might have been overly diagnosed when patients presented with fever and a pattern of pulmonary edema on the chest radiograph.

Septic patients who are presented as myocardial infarction may have altered clinical outcomes. Sepsis is an important etiology of type II MI, which results from increased oxygen demand or decreased myocardium supply. Severe septic patients with obstructive coronary artery disease were associated with a significant mortality increase at 60 days [33]. In this study, the patients with initial blood stream infection on admission were less than 3%. We also identified that cardiogenic factors, respiratory problems and bleeding were the leading causes of cardiac arrests in this study, while sepsis was the least dominant etiology.

The incidence of newly developed heart failure was higher in the hyperthermic group, which was consistent with previous reports that febrile patients were prone to have cardiac pump failure and a higher readmission rate [11]. The effect of the initial body temperature on long-term prognosis requires further investigation and validation.

Inflammation plays a key role in abnormal body temperature. Several medications, such as colchicine, canakinumab, or tocilizumab, which may also alter body temperature or modulate the inflammatory response, have already been evaluated in patients with acute coronary syndrome [34, 35]. However, no significant differences were observed in our study regarding WBC or CRP among the different triage body temperature groups.

Significant differences in several adverse events, but not in-hospital cardiac arrest, were observed among the different triage body temperature groups. Our study showed that age, respiratory rate, heart rate, oxygen saturation and CKD were risk factors for complications in patients with MI. Increased age was correlated with a poor outcome of MI [36, 37]. An initial heart rate greater than 100 beats/min could predict poor left ventricular function [38], while oxygen saturation on admission was related to all-cause mortality [39]. A risk index has been proposed based on age, heart rate and systolic blood pressure to predict mortality in STEMI [40]. Our study identified several key factors of triage vital signs and comorbidities and thus could improve risk stratification in acute MI patients.

The initial body temperature has been frequently utilized to triage patients and predict prognosis in clinical care. It was adopted earlier in the National Early Warning Score and Modified Early Warning Score and later proposed in the Emergency Department Triage Early Warning Score to help allocate clinical resources [41]. A triage body temperature less than 36.5 °C is independently associated with 30-day mortality in patients with hip fracture [42]. Body temperature is negatively correlated with the prognosis of acute stroke [43]. Early detection of coronavirus aided by triage body temperature and other parameters was established during the pandemic [44]. Diagnostic or prognostic predictive models for different diseases that incorporate triage body temperature could be developed with the use of machine learning and artificial intelligence.

### Limitation

This study had several limitations. First, it was a retrospective study. Although there were no significant differences in most basic characteristics, subtle differences in the study population may be undetected. Second, this study was conducted in a single tertiary hospital in Taiwan. Whether the results of our study can be generalized to Asian or other populations need to be investigated. The retrospective design and the limited sample size of this study could render the study results relatively underpowered. We applied the POWER analysis with the software G^*^Power between the groups [25, 45], and a Power (1-β err prob) = 0.877 and a Cohen’s ω = 0.16 were obtained. Considering the Chi-Square test and the Cohen’s ω (0.1 as a small effect, 0.3 as a medium effect, and 0.5 as a large effect), we had a low effect size to draw our conclusions [46]. Third, the cutoff for body temperature in this study was determined according to previous studies. The different cutoffs of body temperature may lead to alterations in statistical analysis. Fourth, menstruation could affect body temperature. Core body temperature is lower in postmenopausal women than in premenopausal women [47]. Finally, most patients in this study were elderly, with an average age of 69.1 years old. Thus, stratified analysis on groups of different ages may help to identify sensitive variates in geriatrics. Our study was to investigate the patients with AMI in the ED and the patient number was rather limited. Although we tried to figure out and adjust the bias and covariates by multivariable logistic regression in the study, it was difficult to be random by matching method in the clinical scenario.

## Conclusion

The triage body temperature of patients who have acute MI could be associated with different clinical presentations. This study investigated normothermic, hypothermic and hyperthermic groups and found differences in biochemical parameters, the length of hospital stay and adverse events but not in cardiac arrest or overall complications. Several risk factors were proposed to help stratify patients and allocate clinical resources. Further studies are warranted to validate the correlation between infarction size and initial body temperature and the correlation of initial body temperature with the long-term outcome of acute MI.

### Electronic supplementary material

Below is the link to the electronic supplementary material.

**Additional File 1: Figure S1**: The distribution of triage body temperature

**Additional File 2: Table 1**: The main causes of in-hospital cardiac arrests

**Additional File 3: Table 2**: Propensity matching with baseline characteristics and comorbidities for patients with IHCA

## Data Availability

The datasets used and analyzed during the current study are available from the corresponding author on reasonable request.

## Abbreviations

AOR: Adjusted odds ratio
BT: Body temperature
CAD: Coronary artery disease
CI: Confidence interval
CKD: Chronic kidney disease
CK: Creatine kinase
CRP: C-reactive protein
CVA: Cerebral vascular accident
DBP: Diastolic blood pressure
DM: Diabetes mellitus
ED: Emergency department
HF: Heart failure
HR: Heart rate
HTN: Hypertension
ICU: Intensive care unit
IL: Interleukin
IQR: Interquartile range
MI: Myocardial infarction
IHCA: In-hospital cardiac arrest
MBP: Mean blood pressure
NSTEMI: Non-ST-elevation myocardial infarction
OR: Odds ratio
PCI: Percutaneous coronary intervention
RR: Respiratory rate
SBP: Systolic blood pressure
STEMI: ST-elevation myocardial infarction
WBC: White blood cell

## References

[1] Kacprzak M, Kidawa M, Zielińska M (2012). Fever in myocardial infarction: is it still common, is it still predictive?. Cardiol J.

[2] Löfmark R, Nordlander R, Orinius E (1978). The temperature course in acute myocardial infarction. Am Heart J.

[3] Nahrendorf M, Pittet MJ, Swirski FK (2010). Monocytes: protagonists of infarct inflammation and repair after myocardial infarction. Circulation.

[4] Dutta P, Courties G, Wei Y, Leuschner F, Gorbatov R, Robbins CS, Iwamoto Y, Thompson B, Carlson AL, Heidt T (2012). Myocardial infarction accelerates atherosclerosis. Nature.

[5] Frangogiannis NG (2014). The inflammatory response in myocardial injury, repair, and remodelling. Nat reviews Cardiol.

[6] Hale SL, Kloner RA (2002). Elevated body temperature during myocardial ischemia/reperfusion exacerbates necrosis and worsens no-reflow. Coron Artery Dis.

[7] Herlitz J, Bengtson A, Hjalmarson A, Wilhelmsen L (1988). Body temperature in acute myocardial infarction and its relation to early intervention with metoprolol. Int J Cardiol.

[8] Ben-Dor I, Haim M, Rechavia E, Murininkas D, Nahon M, Harell D, Porter A, Iakobishvili Z, Scapa E, Battler A (2005). Body temperature - a marker of infarct size in the era of early reperfusion. Cardiology.

[9] Navinan MR, Mendis S, Wickramasinghe S, Kathirgamanathan A, Fernando T, Yudhisdran J (2019). Inflammation in ST- elevation myocardial infarction: risk factors, patterns of presentation and association with clinical picture and outcome, an observational study conducted at the Institute of Cardiology-National Hospital of Sri Lanka. BMC Cardiovasc Disord.

[10] Jang WJ, Yang JH, Song YB, Chun WJ, Oh JH, Park YH, Lee MR, Hwang JK, Hwang JW, Hahn JY et al. Clinical significance of Postinfarct Fever in ST-Segment Elevation myocardial infarction: a Cardiac magnetic resonance imaging study. J Am Heart Assoc. 2017;6(4):e005687. 10.1161/JAHA.117.005687 PMC5533041 28438740

[11] Naito K, Anzai T, Yoshikawa T, Maekawa Y, Sugano Y, Kohno T, Mahara K, Okabe T, Asakura Y, Ogawa S (2007). Increased body temperature after reperfused acute myocardial infarction is associated with adverse left ventricular remodeling. J Card Fail.

[12] Cho HO, Nam CW, Lee HM, Shin HW, Cho YK, Yoon HJ, Park HS, Kim H, Chung IS, Hur SH (2014). Fever after primary percutaneous coronary intervention in ST-segment elevation myocardial infarction is associated with adverse outcomes. Int J Cardiol.

[13] Ramgopal S, Horvat CM, Adler MD (2020). Association of triage hypothermia with in-hospital mortality among patients in the emergency department with suspected sepsis. J Crit Care.

[14] Buse S, Blancher M, Viglino D, Pasquier M, Maignan M, Bouzat P, Annecke T, Debaty G (2017). The impact of hypothermia on serum potassium concentration: a systematic review. Resuscitation.

[15] Dallan LAP, Giannetti NS, Rochitte CE, Polastri TF, San Martin CYB, Hajjar LA, Lima FG, Nicolau JC, Oliveira Mt M (2021). Cooling as an adjunctive therapy to percutaneous intervention in Acute myocardial infarction: COOL-MI InCor Trial. Ther Hypothermia Temp Manag.

[16] Faizi M, Farrier AJ, Venkatesan M, Thomas C, Uzoigwe CE, Balasubramanian S, Smith RP (2014). Is body temperature an independent predictor of mortality in hip fracture patients?. Injury.

[17] Kuo YS, Lu CH, Chiu PW, Chang HC, Lin YY, Huang SP, Wang PY, Chen CJ, Lin IC, Tang JS et al. Challenges of using Instant Communication Technology in the Emergency Department during the COVID-19 pandemic: a Focus Group Study. Int J Environ Res Public Health. 2021;18(23):12463. 10.3390/ijerph182312463 PMC8656867 34886188

[18] Lin YY, Lai YY, Chang HC, Lu CH, Chiu PW, Kuo YS, Huang SP, Chang YH, Lin CH (2022). Predictive performances of ALS and BLS termination of resuscitation rules in out-of-hospital cardiac arrest for different resuscitation protocols. BMC Emerg Med.

[19] Lapum JL, Verkuyl M, Garcia W, St-Amant O, Tan A. Vital Sign Measurement Across the Lifespan – 1st Canadian Edition; 2019.

[20] Bijur PE, Shah PD, Esses D (2016). Temperature measurement in the adult emergency department: oral, tympanic membrane and temporal artery temperatures versus rectal temperature. Emerg Med J.

[21] Mogensen CB, Vilhelmsen MB, Jepsen J, Boye LK, Persson MH, Skyum F (2018). Ear measurement of temperature is only useful for screening for fever in an adult emergency department. BMC Emerg Med.

[22] Kushimoto S, Gando S, Saitoh D, Mayumi T, Ogura H, Fujishima S, Araki T, Ikeda H, Kotani J, Miki Y (2013). The impact of body temperature abnormalities on the disease severity and outcome in patients with severe sepsis: an analysis from a multicenter, prospective survey of severe sepsis. Crit Care.

[23] Thygesen K, Alpert JS, Jaffe AS, Chaitman BR, Bax JJ, Morrow DA, White HD, Group ESCSD (2019). Fourth universal definition of myocardial infarction (2018). Eur Heart J.

[24] Poldervaart JM, Langedijk M, Backus BE, Dekker IMC, Six AJ, Doevendans PA, Hoes AW, Reitsma JB (2017). Comparison of the GRACE, HEART and TIMI score to predict major adverse cardiac events in chest pain patients at the emergency department. Int J Cardiol.

[25] Jensen MM, Kellett JG, Hallas P, Brabrand M (2019). Fever increases heart rate and respiratory rate; a prospective observational study of acutely admitted medical patients. Acute Med.

[26] Maclean D, Griffiths PD, Emslie-Smith D (1968). SERUM-ENZYMES IN RELATION TO ELECTROCARDIOGRAPHIC CHANGES IN ACCIDENTAL HYPOTHERMIA. The Lancet.

[27] Nee PA, Scane AC, Lavelle PH, Fellows IW, Hill PG (1987). Hypothermic myxedema coma erroneously diagnosed as myocardial infarction because of increased creatine kinase MB. Clin Chem.

[28] Glusman A, Hasan K, Roguin N (1990). Contraindication to thrombolytic therapy in accidental hypothermia simulating acute myocardial infarction. Int J Cardiol.

[29] Paal P, Pasquier M, Darocha T, Lechner R, Kosinski S, Wallner B, Zafren K, Brugger H (2022). Accidental hypothermia: 2021 update. Int J Environ Res Public Health.

[30] Hassan AKM, Bergheanu SC, Hasan-Ali H, Liem SS, van der Laarse A, Wolterbeek R, Atsma DE, Schalij MJ, Jukema JW (2009). Usefulness of peak Troponin-T to Predict Infarct size and long-term outcome in patients with First Acute myocardial infarction after primary percutaneous coronary intervention. Am J Cardiol.

[31] Sopena N, Sabrià M, Pedro-Botet ML, Manterola JM, Matas L, Domínguez J, Modol JM, Tudela P, Ausina V, Foz M (1999). Prospective study of community-acquired pneumonia of bacterial etiology in adults. Eur J Clin Microbiol Infect Dis.

[32] Ogawa H, Kitsios GD, Iwata M, Terasawa T (2020). Sputum Gram Stain for Bacterial Pathogen diagnosis in Community-acquired Pneumonia: a systematic review and bayesian Meta-analysis of diagnostic accuracy and yield. Clin Infect Dis.

[33] Allou N, Brulliard C, Valance D, Esteve JB, Martinet O, Corradi L, Cordier C, Bouchet B, Allyn J (2016). Obstructive coronary artery disease in patients hospitalized for severe sepsis or septic shock with concomitant acute myocardial infarction. J Crit Care.

[34] Lüscher TF (2020). The sooner, the better: anti-inflammation in acute myocardial infarction. Eur Heart J.

[35] Wang H, Liu Z, Shao J, Lin L, Jiang M, Wang L, Lu X, Zhang H, Chen Y, Zhang R (2020). Immune and inflammation in Acute Coronary Syndrome: Molecular Mechanisms and therapeutic implications. J Immunol Res.

[36] Mehta Rajendra H, Rathore Saif S, Radford Martha J, Wang Y, Wang Y, Krumholz Harlan M (2001). Acute myocardial infarction in the elderly: differences by age. J Am Coll Cardiol.

[37] Kim JH, Chae S-C, Oh DJ, Kim H-S, Kim YJ, Ahn Y, Cho MC, Kim CJ, Yoon J-H, Park H-Y. Multicenter Cohort Study of Acute Myocardial Infarction in Korea–Interim Analysis of the Korea Acute Myocardial Infarction Registry-National Institutes of Health Registry. Circ J. 2016;80(6):1427–36. 10.1253/circj.CJ-16-0061 27118621

[38] van ‘t Hof AWJ, Rasoul S, van de Wetering H, Ernst N, Suryapranata H, Hoorntje JCA, Dambrink J-HE, Gosselink M, Zijlstra F, Ottervanger JP (2006). Feasibility and benefit of prehospital diagnosis, triage, and therapy by paramedics only in patients who are candidates for primary angioplasty for acute myocardial infarction. Am Heart J.

[39] Yu Y, Wang J, Wang Q, Wang J, Min J, Wang S, Wang P, Huang R, Xiao J, Zhang Y (2020). Admission oxygen saturation and all-cause in-hospital mortality in acute myocardial infarction patients: data from the MIMIC-III database. Ann Transl Med.

[40] Morrow DA, Antman EM, Giugliano RP, Cairns R, Charlesworth A, Murphy SA, de Lemos JA, McCabe CH, Braunwald E (2001). A simple risk index for rapid initial triage of patients with ST-elevation myocardial infarction: an InTIME II substudy. The Lancet.

[41] Lee SB, Kim DH, Kim T, Kang C, Lee SH, Jeong JH, Kim SC, Park YJ, Lim D (2020). Emergency Department Triage early warning score (TREWS) predicts in-hospital mortality in the emergency department. Am J Emerg Med.

[42] Faizi M, Farrier AJ, Venkatesan M, Thomas C, Uzoigwe CE, Balasubramanian S, Smith RP. Is body temperature an independent predictor of mortality in hip fracture patients? Injury. 2014;45(12):1942–5. 10.1016/j.injury.2014.09.024 25458058

[43] Sung S-F, Hung L-C, Hu Y-H (2021). Developing a stroke alert trigger for clinical decision support at emergency triage using machine learning. Int J Med Informatics.

[44] An C, Oh HC, Chang JH, Oh S-J, Lee JM, Han CH, Kim SW (2021). Development and validation of a prognostic model for early triage of patients diagnosed with COVID-19. Sci Rep.

[45] Faul F, Erdfelder E, Lang A-G, Buchner A (2007). G*Power 3: a flexible statistical power analysis program for the social, behavioral, and biomedical sciences. Behav Res Methods.

[46] Serdar CC, Cihan M, Yücel D, Serdar MA (2021). Sample size, power and effect size revisited: simplified and practical approaches in pre-clinical, clinical and laboratory studies. Biochem Med (Zagreb).

[47] Neff LM, Hoffmann ME, Zeiss DM, Lowry K, Edwards M, Rodriguez SM, Wachsberg KN, Kushner R, Landsberg L. Core body temperature is lower in postmenopausal women than premenopausal women: potential implications for energy metabolism and midlife weight gain. Cardiovasc Endocrinol. 2016;5(4):151–4. 10.1097/XCE.0000000000000078 PMC5242227 28111609

