# Supplementary figures and images for: Triage body temperature and its influence on patients with acute myocardial infarction
Source: BMC Cardiovasc Disord. 2023 Aug 4;23:388. doi: 10.1186/s12872-023-03372-y (PMC10403904; doi:10.1186/s12872-023-03372-y)

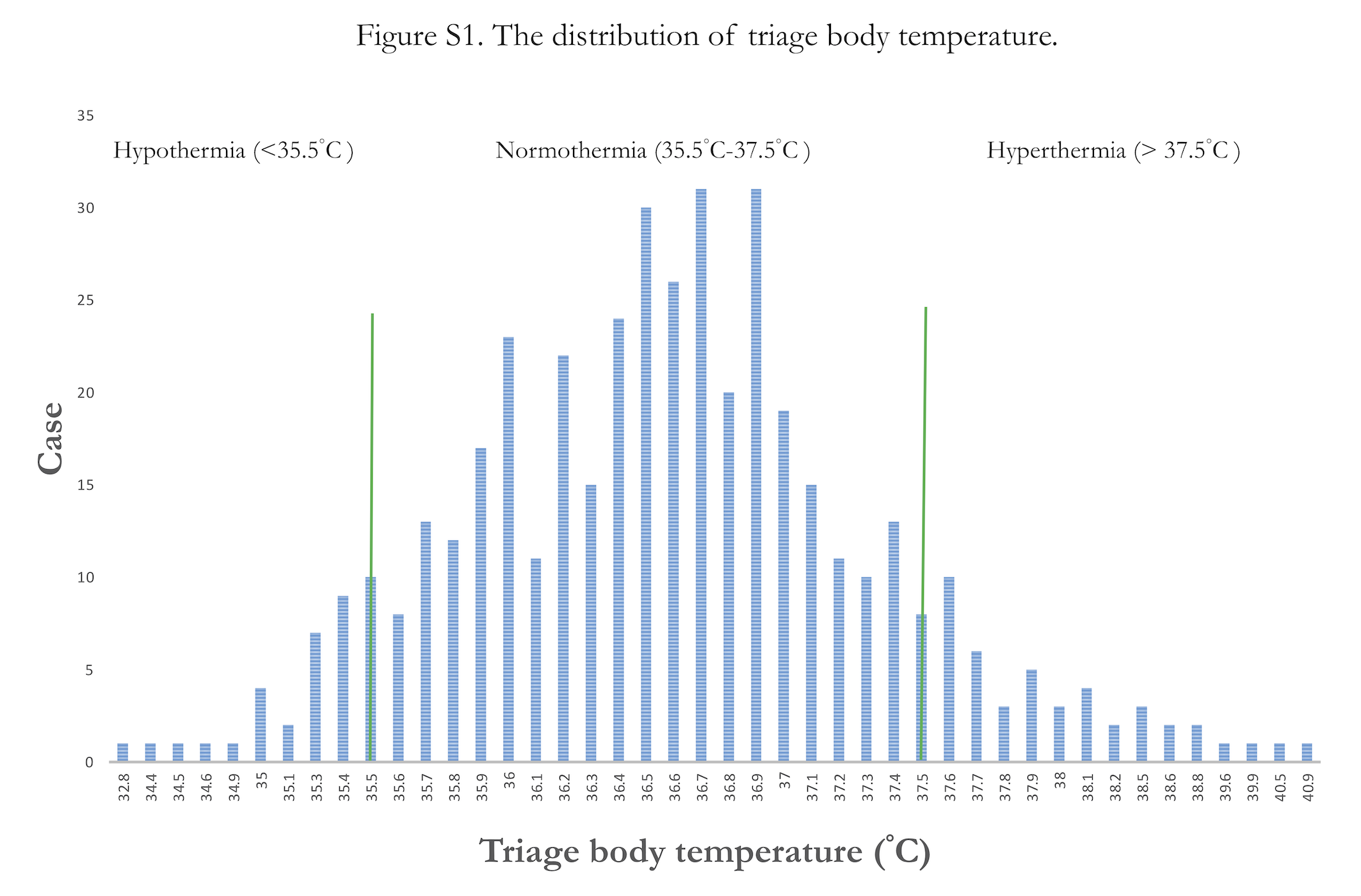

Supplement: Supplementary file 1 — Additional File 1: Figure S1: The distribution of triage body temperature [file 12872_2023_3372_MOESM1_ESM.tiff]
