# Supplementary material for: Triage body temperature and its influence on patients with acute myocardial infarction
Source: BMC Cardiovasc Disord. 2023 Aug 4;23:388. doi: 10.1186/s12872-023-03372-y (PMC10403904; doi:10.1186/s12872-023-03372-y)
Supplement: Supplementary file 2 — Additional File 2: Table 1: The main causes of in-hospital cardiac arrests [file 12872_2023_3372_MOESM2_ESM.docx]

Supplementary Table 1: The main causes of in-hospital cardiac arrests.

| Cardiac arrest  (N=37) | Normothermic  (N=28)  n (%) | Hypothermic  (N=4)  n (%) | Hyperthermic  (N=5)  n (%) | p value |
| --- | --- | --- | --- | --- |
| Cardiogenic | 21 (75%) | 3 (75%) | 3 (60%) | 0.781 |
| Respiratory | 7 (25%) | 2 (50%) | 0 (0%) | 0.218 |
| Sepsis | 2 (7.1%) | 0 (0%) | 1 (20%) | 0.512 |
| Bleeding | 4 (14.3%) | 0 (0%) | 1 (20%) | 0.664 |

Note: The main causes of in-hospital cardiac arrest could be more than one for each patient.
