# Supplementary material for: Triage body temperature and its influence on patients with acute myocardial infarction
Source: BMC Cardiovasc Disord. 2023 Aug 4;23:388. doi: 10.1186/s12872-023-03372-y (PMC10403904; doi:10.1186/s12872-023-03372-y)
Supplement: Supplementary file 3 — Additional File 3: Table 2: Propensity matching with baseline characteristics and comorbidities for patients with IHCA [file 12872_2023_3372_MOESM3_ESM.docx]

Supplementary Table 2. Propensity matching with baseline characteristics and comorbidities for patients with IHCA.

|  | AOR (95% CI)^#^ | AOR (95% CI)^$^ |
| --- | --- | --- |
| Age (y/o) | 1.03 (1.00-1.07)* | 0.67 (0.54-0.84)*** |
| Triage BT (*°*C) |  |  |
| Normal | 1.00 | 1.00 |
| Hypothermia | 2.17 (0.59-7.89) | 2.23 (0.00-56372.24) |
| Fever | 0.98 (0.30-3.26) | 0.21 (0.02-2.52) |
| MBP (mmHg) | 0.97 (0.95-0.98)*** | 0.93 (0.88-0.99)* |
| DM | 2.11 (0.95-4.73) | 40.73 (3.62-458.89)** |
| PCI | 0.24 (0.09-0.61)** | 0.03 (0.00-0.56)** |
| STEMI | 1.96 (0.83-4.64) | 5.59 (0.53-59.62) |
| NSTEMI | 0.51 (0.22-1.20) | 0.40 (0.18-0.92) |

*p<0.05, **p<0.01, ***p<0.001 / ^a^ single variable, ^b^ full model, ^c^ final model.

^#^ before IHCA matching.

^$^ after IHCA matching by sex, age, and DM.

Abbreviations: AOR, adjusted odds ratio; BT, body temperature; CI, confidence interval; DM, diabetes mellitus; IHCA, in-hospital cardiac arrest; MBP, mean blood pressure; NSTEMI, non-ST-elevation myocardial infarction; PCI, percutaneous coronary intervention; STEMI, ST-elevation myocardial infarction.
